# Efficacy of carboprost tromethamine combined with leonurus japonicus for prevention of postpartum hemorrhage in high-risk pregnant women: A protocol for systematic review and meta-analysis

**DOI:** 10.1097/MD.0000000000026792

**Published:** 2021-07-30

**Authors:** Fang Zong, Yanmin Cao

**Affiliations:** Department of Obstetric, Cangzhou Central Hospital, Hebei, China.

**Keywords:** carboprost tromethamine, leonurus japonicus injection, meta-analysis, postpartum hemorrhage, protocol

## Abstract

**Background:**

No well-designed and systematic evaluation of the efficacy and safety of leonurus japonicus injection (LJI) in combination with carboprost tromethamine has been found. Therefore, we undertook a meta-analysis to assess the efficacy and safety of carboprost tromethamine combined with LJI for the prevention of postpartum hemorrhage in high-risk pregnant women to provide new evidence-based medical evidence for clinical treatment.

**Methods:**

This systematic review and meta-analysis would be performed according to Preferred Reporting Items for Systematic reviews and Meta-Analyses guidelines. The following databases including EMBASE, MEDICINE, Wanfang, China National Knowledge Infrastructure database, and Cochrane central controlled trial registries were searched by 2 reviewers from inception to July 2021. Mesh and keyword search terms were “motherwort,” “Yimucao,” “leonurus japonicas,” “carboprost tromethamine,” and “postpartum hemorrhage.” Any cohort studies that assessed the efficacy and safety of carboprost tromethamine combined with LJI for the prevention of postpartum hemorrhage would be included. *P* < .05 was set as the level of significance.

**Results:**

The review would add to the existing literature by showing compelling evidence and improved guidance in clinic settings.

**OSF registration number:**

10.17605/OSF.IO/2WC53.

## 1 Introduction

Despite great efforts to reduce maternal mortality, postpartum hemorrhage remains the largest direct cause of maternal mortality, accounting for nearly a quarter of all deaths worldwide, and contributes to long-term disability and severe maternal morbidity, such as blood transfusions, emergency surgery, and admission to the intensive care unit.^[1]^ The American College of Obstetricians and Gynecologists defines early postpartum hemorrhage as total blood loss associated with signs and symptoms of hypovolemia of at least 1000 mL within 24 hours after delivery.^[2]^ Although maternal mortality has become a rare event in developed countries, it remains an important indicator of the quality of obstetric care and the health status of mothers.

Leonurus japonicus (motherwort) has been widely used to prevent postpartum hemorrhage in China since 1972. It is a traditional Chinese medicine commonly used to treat gynecological diseases in China for thousands of years. According to relevant studies, leonurus japonicus injection (LJI) is effective in the inferior uterine segment without a receptor saturation effect, which reduces the risk of adverse events caused by excessive oxytocin use.^[3–5]^ In addition, LJI usually works by intramuscular injection when cold storage and infusion are not convenient. Carbprost tromethamine, a synthetic 15-methyl analogue of prostaglandin F2α, has been reported to be 84% to 96% effective in the treatment of persistent hemorrhage due to uterine dystonic dysfunction. However, since its introduction, there have been few studies on the prevention and treatment of postpartum hemorrhage.^[6,7]^

Given that the routine practice of using LJI is widespread in China, studies addressing the effects of LJI have been accumulating over the past few years. Most clinical trials and experience indicate that prophylactic use of LJI alone or in combination with carboprost tromethamine may have a good effect on the prevention of postpartum hemorrhage after delivery.^[3,8,9]^ However, no well-designed and systematic evaluation of the efficacy and safety of LJI in combination with carboprost tromethamine has been found. Therefore, we undertook a meta-analysis to assess the efficacy and safety of carboprost tromethamine combined with LJI for the prevention of postpartum hemorrhage in high-risk pregnant women to provide new evidence-based medical evidence for clinical treatment.

## 2 Materials and methods

### 2.1 Searching strategy

The following databases including EMBASE, MEDICINE, Wanfang, China National Knowledge Infrastructure database, and Cochrane central controlled trial registries were searched by 2 reviewers from inception to July 2021. Mesh and keyword search terms were “motherwort,” “Yimucao,” “leonurus japonicas,” “carboprost tromethamine,” and “postpartum hemorrhage.” We also searched clinicaltrial.gov and the China ClinicalTrial Registry website to identify other eligible clinical trials. The reference lists of included studies were searched for additional eligible study. There were no restrictions in terms of language. This systematic review and meta-analysis would be performed according to Preferred Reporting Items for Systematic reviews and Meta-Analyses guidelines. The prospective registration has been approved by the Open Science Framework registries. Ethical approval was not necessary because the present meta-analysis would be performed based on previously published studies.

### 2.2 Eligibility criteria

Study included in this review had to meet all of the following inclusion criteria in the PICOS order:

1. population: high-risk pregnant women;
2. intervention group (group 1): patients with carboprost tromethamine combined with LJI;
3. comparison group (group 2): patients with placebo or no carboprost tromethamine combined with LJI;
4. outcome measures: blood loss within 2 hours, blood loss within 24 hours, postpartum hemorrhage, and adverse events.
5. study design: cohort studies.

Biomechanical studies, in vitro studies, review articles, techniques, case reports, letters to the editor, and editorials were excluded.

### 2.3 Data extraction

The data was extracted in duplicate. The reasons of exclusion at this stage were summarized. Results were recorded on trial data extraction forms and Excel spreadsheets. Data extracted related to: country and study date; participants (indication, age, sex); inclusion and exclusion criteria; intervention content and control group; setting, timing, duration, and intensity of the intervention; follow-up time; subsequent losses and their causes; and the outcomes. For the results reported as continuous variables, the mean and standard deviation were extracted. If the results were reported as mean and confidence intervals, or median and quartile spacing, the appropriate conversion would be applied. If necessary, the lead author of the study would be contacted for missing data. We also asked whether any results not reported in their publications had been collected. If the author had provided information to other reviewers, the data would be included in our analysis and acknowledged appropriately.

### 2.4 Statistical analysis

The present study would be performed by Review Manager Software (RevMan Version 5.3, The Cochrane Collaboration, Copenhagen, Denmark). Risk ratios with a 95% confidence interval or mean difference with 95% confidence interval were assessed for dichotomous outcomes or continuous outcomes, respectively. *P* < .05 was set as the level of significance. It would also be considered statistically significant if “1” was not included in the 95% confidence interval of risk ratios or “0” was not included in the 95% confidence interval of mean difference. The *Q* test and *I*^2^ statistic were used to assess the heterogeneity. When *I*^2^ < 40%, it was considered to represent no significant heterogeneity, and then the fixed-effect model was used. On contrary, a random effects model was used for the heterogeneity if *I*^2^ ≥ 40%. We also conducted the sensitivity analysis to evaluate whether any single study had the weight to skew on the overall estimate and data. The *Z* test was used to assess the overall effect.

### 2.5 Assessments of study quality

Two of us independently assessed the risk of bias in the included studies using parameters defined in the Cochrane Handbook for Systematic Reviews of Interventions criteria. Differences were resolved through discussion and consensus among reviewers. Based on the information provided by the included studies, each item was recorded as high risk of bias, low risk of bias, or unclear. Two reviewers independently assessed the quality of the body of evidence for different outcomes using the Grades of Recommendation, Assessment, Development, and Evaluation approach, a proven and widely practiced tool for assessing the quality of scientific evidence. Based on the Grades of Recommendation, Assessment, Development, and Evaluation approach, we assessed 5 areas, ranking the strength of evidence for each result.

## 3 Discussion

Given that the routine practice of using LJI is widespread in China, studies addressing the effects of LJI have been accumulating over the past few years. Most clinical trials and experience indicate that prophylactic use of LJI alone or in combination with carboprost tromethamine may have a good effect on the prevention of postpartum hemorrhage after delivery.^[3,8,9]^ However, no well-designed and systematic evaluation of the efficacy and safety of LJI in combination with carboprost tromethamine has been found. Therefore, we undertook a meta-analysis to assess the efficacy and safety of carboprost tromethamine combined with LJI for the prevention of postpartum hemorrhage in high-risk pregnant women to provide new evidence-based medical evidence for clinical treatment.

## Author contributions

**Conceptualization:** Yanmin Cao.

**Data curation:** Fang Zong, Yanmin Cao.

**Formal analysis:** Fang Zong.

**Funding acquisition:** Yanmin Cao.

**Investigation:** Fang Zong, Yanmin Cao.

**Methodology:** Fang Zong.

**Project administration:** Yanmin Cao.

**Resources:** Yanmin Cao.

**Software:** Fang Zong.

**Supervision:** Yanmin Cao.

**Validation:** Fang Zong.

**Writing – original draft:** Fang Zong.

**Writing – review & editing:** Yanmin Cao.
